# Perovskite-based electrocatalysts for oxygen evolution reaction in alkaline media: A mini review

**DOI:** 10.3389/fchem.2022.1024865

**Published:** 2022-10-07

**Authors:** Dongkyu Kim, Lee Seul Oh, Jong Hyeok Park, Hyung Ju Kim, Seonggyu Lee, Eunho Lim

**Affiliations:** ^1^ Chemical & Process Technology Division, Korea Research Institute of Chemical Technology (KRICT), Daejeon, South Korea; ^2^ Department of Chemical and Biomolecular Engineering, Yonsei University, Seoul, South Korea; ^3^ Advanced Materials and Chemical Engineering, University of Science and Technology (UST), Daejeon, South Korea; ^4^ Department of Chemical Engineering, Kumoh National Institute of Technology (KIT), Gumi, South Korea; ^5^ Department of Energy Engineering Convergence, Kumoh National Institute of Technology (KIT), Gumi, South Korea

**Keywords:** water electrolysis, oxygen evolution reaction, electrocatalysts, metal oxides, perovskites

## Abstract

Water electrolysis is one of the attractive technologies for producing clean and sustainable hydrogen fuels with high purity. Among the various kinds of water electrolysis systems, anion exchange membrane water electrolysis has received much attention by combining the advantages of alkaline water electrolysis and proton exchange membrane water electrolysis. However, the sluggish kinetics of the oxygen evolution reaction, which is based on multiple and complex reaction mechanisms, is regarded as a major obstacle for the development of high-efficiency water electrolysis. Therefore, the development of high-performance oxygen evolution reaction electrocatalysts is a prerequisite for the commercialization and wide application of water electrolysis systems. This mini review highlights the current progress of representative oxygen evolution reaction electrocatalysts that are based on a perovskite structure in alkaline media. We first summarize the research status of various kinds of perovskite-based oxygen evolution reaction electrocatalysts, reaction mechanisms and activity descriptors. Finally, the challenges facing the development of perovskite-based oxygen evolution reaction electrocatalysts and a perspective on their future are discussed.

## Introduction

Hydrogen is considered as a clean and sustainable energy source that can replace the fossil fuel-based energy currently in use (Momirlan and Veziroglu, 2002; Wang et al., 2019). There are several methods to produce hydrogen, but the most of hydrogen is currently produced by a gas reforming process that has the disadvantage of producing carbon dioxide (Han et al., 2020; Kwon et al., 2020; Tian et al., 2020). In order to overcome this disadvantage, water electrolysis has received much attention as an environmentally friendly method for hydrogen production (Xie et al., 2019; Huang et al., 2020; Tian et al., 2021).

**FIGURE 1:**
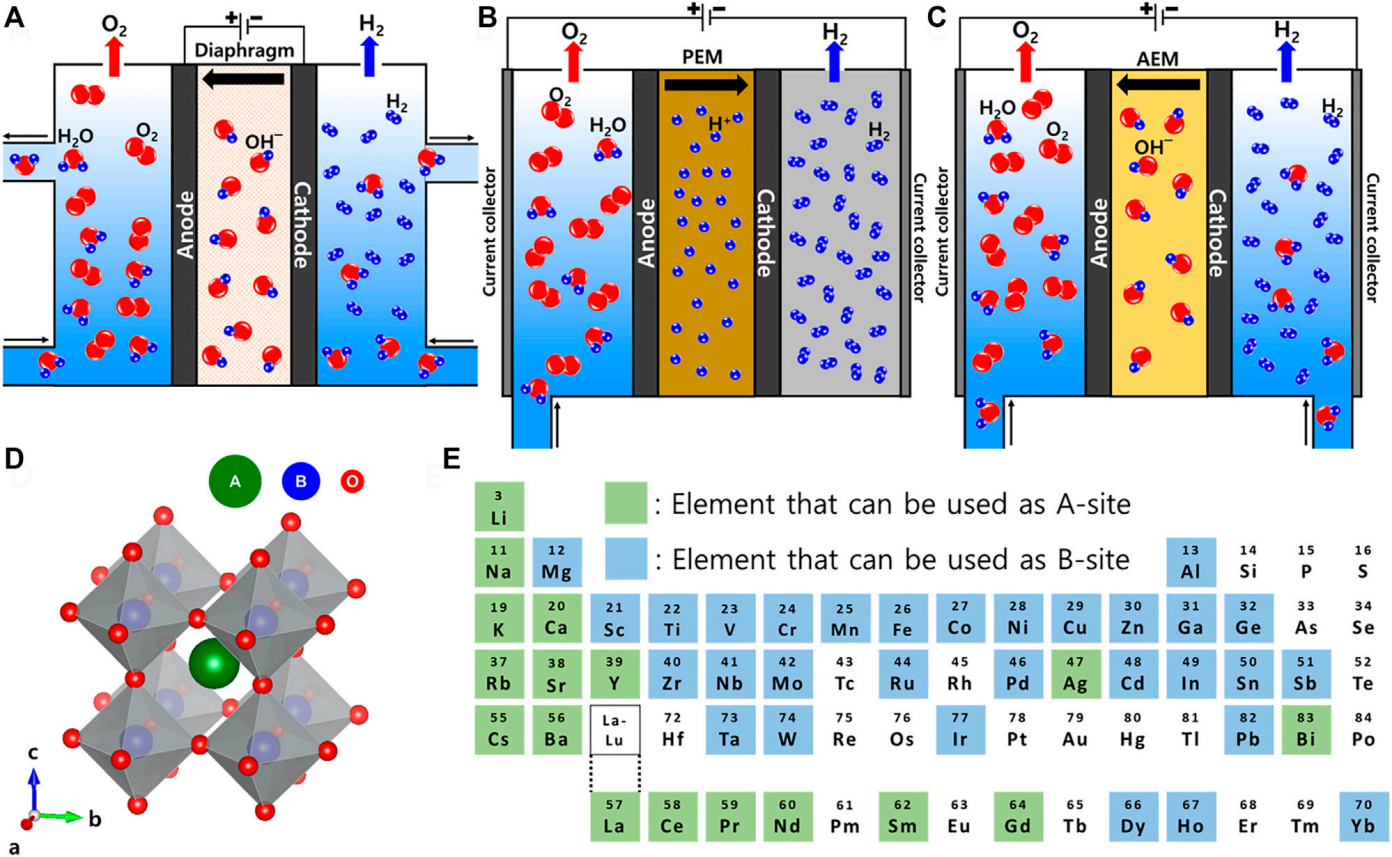
Schematic illustration of **(A)** AWE, **(B)** AEMWE, and **(C)** PEMWE systems. **(D)** Scheme of typical ABO_3_ perovskite structure. **(E)** A map of the elements in the periodic table that can be used for A- and B-sites of the ABO_3_ perovskite structure.

The water electrolysis system can be roughly classified into three types according to the pH of the electrolyte and the system configuration (Figures 1A–C) (Li et al., 2020). Alkaline water electrolysis (AWE) is composed of inexpensive transition metal electrocatalysts (Figure 1A), and generally operates in a high concentration alkaline electrolyte (e.g., 20%–40% KOH) (Liu et al., 2020; Jang et al., 2021; Yang et al., 2021). AWE is a well-established mature technology and has been commercialized with a wide range of applications. However, AWE has the disadvantage of low energy efficiency and current density (0.2–0.4 A cm^−2^ in a voltage range of 1.8–2.4 V) (Carmo et al., 2013; Yu et al., 2018). In addition, undesired gas crossover and/or corrosion of components by the concentrated alkaline electrolyte can occur (Solmaz and Kardaş, 2009; Ahn et al., 2014).

Proton exchange membrane water electrolysis (PEMWE) is one of the promising technologies that will appear in the near future (Figure 1B) (Jang et al., 2021). PEMWE can produce high-purity hydrogen with high energy efficiency and current density (0.6–2.0 A cm^−2^ at a voltage of 1.8–2.2 V) (Carmo et al., 2013; Manna et al., 2021). The design and operating conditions of PEMWE can be easily controlled. Although recent efforts are being made to produce hydrogen using PEMWE, it still requires a large amount of noble metal-based electrocatalysts (e.g., Pt and Ir) in both electrodes (Lee et al., 2020; Oh et al., 2021), and the oxygen evolution reaction (OER) electrocatalysts suffers from dissolution at a high operating voltage with an acidic environment. Thus, the high cost and low durability of the system lower the economic efficiency and hinder the wide application of PEMWE (Kwon et al., 2020).

Anion exchange membrane water electrolysis (AEMWE) is attracting attention as a system for hydrogen production that combines the advantages of AWE and PEMWE (Figure 1C) (Shirvanian et al., 2021). Since the system mainly operates in a low concentration alkaline electrolyte (e.g., 0.1–1.0 M KOH), high-purity hydrogen can be produced using inexpensive transition metal-based electrocatalysts with high current density (0.2–1.4 A cm^−2^ at the voltage of 1.6–1.8 V) (Kim et al., 2015; Lee et al., 2021; Yang et al., 2021). However, AEMWE is still in the early stages of research and there are many problems to be solved, such as low electrocatalytic activity and ionic conductivity.

Although the theoretical voltage required for the water electrolysis system is 1.23 V (Kauffman et al., 2016; Chang et al., 2020), the overpotential results from variable resistance elements such as kinetic and ohmic losses (Rheinländer and Durst, 2021). Mainly because the reaction rate and complicated reaction path of the OER that is based on a 4-electron reaction than that of the hydrogen evolution reaction (HER), which is based on a 2-electron reaction, the overpotential for kinetic loss mainly occurs in the OER at the anode (Xie et al., 2019; Youk et al., 2019; Park et al., 2020; Xia et al., 2020). Therefore, in order to improve the efficiency of the water electrolysis system, it is very important to reduce the overpotential through the development of a high-performance electrocatalyst in the OER.

In AEMWE, inexpensive transition metal-based electrocatalysts such as Ni, Co, Fe, and Mn have been attracting attention as OER electrocatalysts (Kim et al., 2015; Kauffman et al., 2019; Zhu et al., 2020b; Deng et al., 2020; Teng et al., 2020; Diao et al., 2021; Huang et al., 2022), and various kinds of transition metal-based OER electrocatalysts such as metal (oxy)hydroxides (Ahn et al., 2022; Li et al., 2022), spinel (Maiyalagan et al., 2014; Bao et al., 2015) and perovskite (Fabbri et al., 2017; Zhu et al., 2017; Ramesh et al., 2018; Guo et al., 2019) structured metal oxides have been widely investigated. Among these electrocatalysts, perovskite-based metal oxides provide improved OER activities, mainly due to the many advantages resulting from various transition metal combinations, defect engineering, etc. (Wang et al., 2019; She et al., 2019; Zhang et al., 2019; Antipin and Risch, 2020; Zhu et al., 2021a; Zhu et al., 2021b; She et al., 2021). In this mini review, we focus on the recent research trends of perovskite as an electrocatalyst in the OER and discuss the reaction mechanisms and activity descriptors.

## Research on perovskite OER electrocatalysts

### Characteristics of perovskites

Perovskite is a type of metal oxide that has the chemical structure of ABO_3_ (Figure 1D) (Jonker and Van Santen, 1950; Nkwachukwu and Arotiba, 2021). Rare Earth or alkaline Earth metals, which have a relatively large ionic radius, are in the A-site, and transition metals, which have a relatively small ionic radius, are in the B-site (Yoon et al., 2014a). The stability and distortion of the perovskite crystal structures in various combinations can be defined by considering Goldschmidt’s tolerance factor (*t*) (Nkwachukwu and Arotiba, 2021).

$$t=\frac{r_{A}+r_{O}}{\sqrt{2}\left(r_{B}+r_{O}\right)}$$ (1)

In Eq. 1, *r*_A_, *r*_B_, and *r*_O_ are the ionic radius of A, B cations, and anions (usually oxygen), respectively. If the value of *t* is between 0.9-1, the structure of the perovskite has an ideal cubic structure, but if the value of *t* is between 0.71 and 0.9, it has an orthorhombic or rhombohedral structure (Li et al., 2016). Therefore, according to each ionic radius, the elements that can be used at each perovskite metal site are determined. La, Sr, Ba, Ca, etc. are mainly used for the A-site, and Cr, Mn, Fe, Co, Ni, etc. are mainly used for the B-site (Figure 1E) (Sun and Zhou, 2021). Various combinations of perovskite are possible from these diverse A- and B-site metal cations, and moreover partial substitution of a metal cation is possible for each site (e.g., A_x_A′_1-x_BO_3-δ_ and AB_y_B′_1-y_O_3-δ_) (Yoon et al., 2014b; She et al., 2022). Therefore, infinitely many new perovskite OER electrocatalysts can be developed through various metal combinations in the future.

### Oxygen evolution reaction mechanisms for perovskites in alkaline media

**FIGURE 2:**
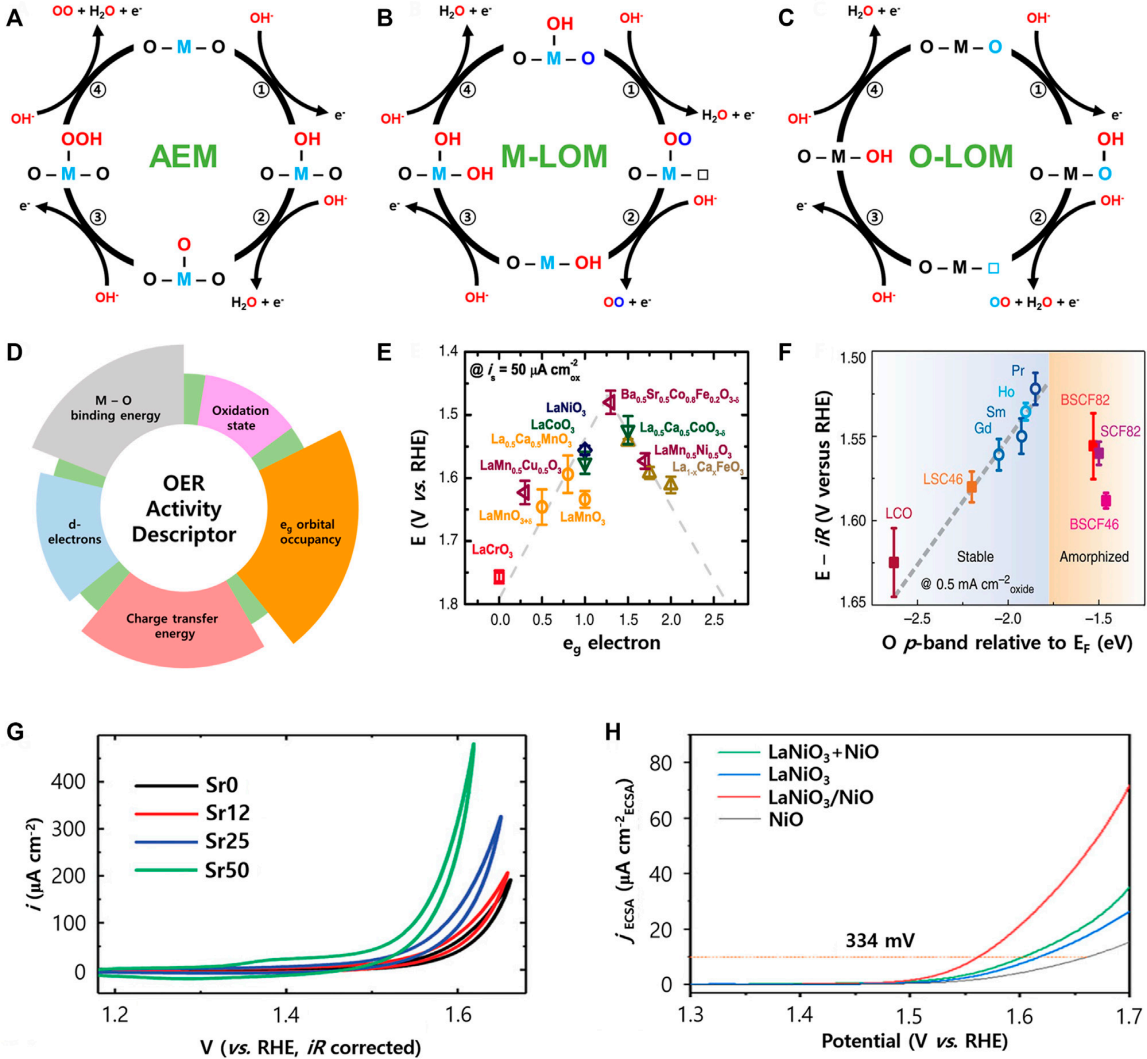
Schematic illustration of OER mechanisms involving **(A)** AEM, **(B)** M-LOM, and **(C)** O-LOM. **(D)** Various descriptors related to the perovskite OER activity. **(E)** The relation between the OER activity and the e_g_ orbital occupancy, reproduced with permission from (Suntivich et al., 2011). **(F)** The trend of the *iR*-corrected potential vs. the O *p*-band centre relative to *E*_F_ (eV), reproduced with permission from (Grimaud et al., 2013). **(G)** Cyclic voltammetry data of LSNO films normalized by the specific area, reproduced with permission from (Liu et al., 2019). **(H)** LSV curves of various LaNiO_3_ electrocatalysts normalized by ECSA, reproduced with permission from (Wei et al., 2022).

Research on the OER mechanisms of perovskite electrocatalysts operating in alkaline media has been actively conducted to date (Rossmeisl et al., 2007; Grimaud et al., 2017; Yoo et al., 2018). In general, the OER occurs via an adsorbate evolution mechanism (AEM). Firstly, OH is adsorbed to the active site (transition metal site) of perovskite, and the O intermediate is produced by deprotonation of OH. Then OOH is generated by OH adsorption at the O site. Finally, O_2_ is generated by the second deprotonation of OOH (Figure 2A). In AEM, the minimum overpotential is theoretically limited to 0.3–0.4 V due to the scaling relationship between the adsorption energies of the intermediates. Recently, it was reported that some perovskite OER electrocatalysts delivered overpotentials lower than the theoretical overpotential. Therefore, it was recognized that a new OER mechanism may exist, which led to the discovery of a new OER mechanism called the lattice oxygen oxidation mechanism (LOM).

A key feature of the LOM is the participation of lattice oxygen in the perovskite for the OER, which was revealed by DFT calculations and isotope experiments (Wang et al., 2020). Previous reports classified the LOM as the M-LOM (metal is an active site) and O-LOM (lattice oxygen is an active site) depending on the active site of perovskite. In the M-LOM, OH is first adsorbed at the metal site of perovskite, followed by deprotonation of OH to form an OO intermediate with lattice oxygen. In this process, the lattice oxygen vacancy is formed and this vacancy is in an unstable state. Therefore, the OH fills the vacancy site and oxygen is produced. After that, another OH is bonded to the metal site again, and then deprotonation occurs. Finally, the active site of the perovskite electrocatalyst returns to its initial form and the reaction continues (Figure 2B).

Shao-Horn et al. proposed that the active site in perovskite can be the lattice oxygen site rather than the metal site in the O-LOM (Figure 2C) (Rao et al., 2020). In the O-LOM, the OH is adsorbed to the activated lattice oxygen site to form OOH. Next, the OO is produced by deprotonation of OOH. This process releases O_2_ from the structure and creates oxygen vacancies in its place. Then OH fills the oxygen vacancy site and deprotonation occurs. Therefore, the OER mechanism between the M-LOM and the O-LOM is clearly different owing to the different active sites of the perovskite. In addition, it should be noted that a rational design of the perovskite electrocatalysts for using the LOM is very important because it is more advantageous for enhancing OER activity compared to the AEM.

### Oxygen evolution reaction activity descriptors for perovskites

The activity of the OER can be explained from various descriptors that are factors for the material properties related to OER activity (Figure 2D). To take advantage of the OER mechanism of perovskite, researchers have proposed several descriptors for electrocatalyst design. Bockris et al. (Bockris and Otagawa, 1984) first reported that the OER performance of LaMO_3_ (M = transition metals) was related to the 3d electron number of bulk transition metal ions.

Afterwards, Nørskov et al. (Man et al., 2011) reported that the activity of various perovskite OER electrocatalysts can be predicted by expressing the correlation between the B-site transition metal and oxygen binding energy in a volcano plot using ${∆G}_{O^{*}}^{0}-{∆G}_{{OH}^{*}}^{0}$ . This is because given a constant difference between HOO and HO adsorption energy levels, the overpotential (*η*_*OER*_) is determined by the O adsorption energy. The perovskite on the left branch of the volcano plot indicates that the OER is limited by the O → HOO step due to the strong oxygen adsorption energy. In contrast, the perovskite on the right branch indicates that the OER is limited by the HO → O step due to the weak oxygen adsorption energy. Although the adsorption energy is a powerful descriptor in explaining the activity trend of known perovskites, a more intuitive descriptor is required to develop new perovskite electrocatalysts.

Shao-Horn et al. (Suntivich et al., 2011) reported that e_g_ orbital occupancy of the surface transition metal in perovskite can have a significant effect on OER performance. This is because the e_g_ orbital of the surface transition metal ions directly interacts with oxygen intermediates adsorbed on the metal surface through *σ* bonding. The OER activities of the perovskites showed a volcano plot with the occupancy of the e_g_ orbital, and the electrocatalysts have better activity when the number of e_g_ electron is close to 1 (Figure 2E).

The metal d-band center and oxygen p-band center can also be activity descriptors for the OER (Grimaud et al., 2013). The band center energy level is closely related to the adsorption energy of the intermediates, and the energy difference between the center of the M nd and O 2p bands can change the metal-oxygen hybridization and charge transfer barrier between the transition metal and oxygen. In addition, the position of the O 2p band center relative to the Fermi level can be directly used to predict the performance of the perovskite electrocatalysts. If the O 2p band center is close to the Fermi level, oxygen vacancies are generated and metal-oxygen covalency is increased, resulting in improved OER performance. However, if the O 2p band center becomes too close to the Fermi level, the activity and stability are decreased by the rapid amorphization in the near surface. Therefore, the perovskite electrocatalyst has high activity and stability when the O 2p band center is neither too far nor too close to the Fermi level (Figure 2F).

### Recent development of perovskites for oxygen evolution reaction

There are various methods for synthesizing perovskites, such as solid-state, citric acid assisted sol-gel, hydrothermal, and electrospinning (Jin et al., 2013; Kim et al., 2017; Zhen et al., 2017; Sun et al., 2019). The solid-state reaction method is the most widely used synthetic method for perovskites based on high temperature calcination. However, the synthesized perovskites by solid-state reaction method exhibited extremely low surface area due to the high temperature. To overcome this disadvantage, the researchers employed the citric acid as a complexion agent to chelate with metal cations, and thereby reduce the synthetic temperature. Therefore, the high surface area from porous nanostructure can be achievable in perovskites synthesized by the method. In addition to this, substantial efforts have been devoted to develop the appropriate synthesis methods for OER electrocatalysts. Furthermore, many researchers conducted various studies to improve activity and stability through substitution at the A-site or B-site (to change composition, and defect engineering), morphology/nano engineering and facet control, crystal structure change, complex perovskite, and perovskite hybrid through various synthetic methods.

Du et al. (Liu et al., 2019) suggested that the OER activity was improved by substituting Sr for the A-site of LaNiO_3_. The synthesized sample was named La_1-x_Sr_x_NiO_3_ (LSNO), and when x = 0.5, the current value at 1.6 V (vs. RHE) was 5 times higher than that of the pristine LaNiO_3_ (Figure 2G). The higher Ni oxidation state induced by the substitution of Sr resulted in the increase of Ni 3d-O 2p hybridization. The increase in hybridization causes an increase of the O 2p band upward and a move closer to the E_F_, which as a result increases the OER activity from the promoted electron transfer in oxygen. Li et al. Sun et al. (2020) reported that V-doped LaCoO_3_ can improve electrocatalytic activity and stability by d-band center location for optimized intermediate adsorption. The LaCo_0.8_V_0.2_O_3_ required only a 306 mV overpotential to reach 10 mA cm^−2^ (LaCoO_3_ was 430 mV) in 1.0 M KOH, and the Tafel slope was only 49 mV dec^−1^. Therefore, the A- or B-site substitution strategy in perovskite affects the adsorption energy change of the OER intermediate, which is helpful in improving the OER performance.

Zhou et al. (Li et al., 2021) synthesized ultra-thin LaMnO_3_ nanosheets with different crystal structures (orthogonal, tetragonal, and hexagonal). The orthogonal LaMnO_3_ nanosheets (o-LMON) had the smallest overpotential at 10 mA cm^−2^_disk_, and the onset potential was also 200 mV smaller than that of IrO_2_. In addition, the specific activity calculated by the BET surface area was almost 10 times higher than that of IrO_2_. If the center of the d-band is too far/close to the E_F_, the binding of the oxygenated adsorbed species is too weak/strong, and it may interfere with the adsorption/desorption process, thereby reducing OER activity. o-LMON was evaluated to have the best activity because it is an electrocatalyst in the optimal state for this adsorption/desorption process. Shao et al. (Dai et al., 2019) synthesized 3D ordered macroporous structured LaFe_0.8_Co_0.2_O_3_ (3DOM-LFC82). In 0.1 M KOH solution, the mass activity of 3DOM-LFC82 was 44 A g_ox_^−1^ while that of bulk LaFeO_3_ was only 13 A g_ox_^−1^ at 400 mV of overpotential. This result indicates that a high surface area and good charge and mass transport ability can improve OER activity of the electrocatalyst.

The effect of surface engineering on OER catalytic activity has also received much attention. Chueh et al. (Baeumer et al., 2021) reported that the surface termination of LaNiO_3_ can be well controlled with Ni, which creates excellent OER performance. Therefore, LaNiO_3_ is still attracting attention as a perovskite electrocatalyst with appropriate surface engineering for an alkaline OER with superior activity. Zhao et al. (Zhao et al., 2019) synthesized an amorphous layer on the surface of La_0.8_Sr_0.2_Co_0.8_Fe_0.2_O_3-δ_ (LSCF-2) by the surface reconstruction. LSCF-2 provided 248 mV of overpotential at 10 mA cm^−2^ in 1.0 M KOH. The Tafel slope was 51 mV dec^−1^, and it showed better stability than the RuO_2_ electrocatalyst. It has been reported that this activity and stability are due to the improved ionic conductivity as the oxidation state of Co is changed during the surface reconstruction process.

Recently, a method for constructing the artificial heterostructure of synthesized perovskite in a different way has been studied. OER activity and stability can be improved by heat-treated perovskite in a reductive atmosphere at high temperature to exsolve the B-site transition metal from the perovskite structure. Yan et al. (Wei et al., 2022) reported that LaNiO_3_ could intentionally create a La deficiency and thereby NiO could be exsolved from the parent matrix to form an interface between the perovskite and the NiO, thereby resulting in enhanced OER activity (Figure 2H). The exsolved NiO at the interface was converted to a NiOOH layer during the OER, and exhibited dynamic surface evolution characteristics. Therefore, formation of the interface achieves greater structural flexibility by optimizing the O 2p level of the electrocatalyst, which promotes surface reconstitution with the highly active NiOOH phase to improve OER activity and stability.

In addition, Ruddlesden-Popper (RP) electrocatalysts, which are a type of perovskite structure, have received a lot of attention (Zhu et al., 2020a; Zhu et al., 2020c). Wang et al. synthesized RP Sr_3_(Co_0.8_Fe_0.1_Nb_0.1_)_2_O_7-δ_ (RP-SCFN), and reported that it exhibited improved OER activity with overpotential of 334 mV to reach 10 mA cm^−2^ at 0.1 M KOH. They suggested that the high Co^4+^ content of RP-SCFN resulted in a high covalent oxide, induced high activity as the center of the O 2p band approached the Fermi level. Furthermore, they synthesized an RP/perovskite hybrid electrocatalyst (RP/P-LSCF) composed of a main RP phase (LaSr_3_Co_1.5_Fe_1.5_O_10-δ_, RP-LSCF) and second perovskite phase La_0.25_Sr_0.75_Co_0.5_Fe_0.5_O_3-δ_ (P-LSCF), which has an overpotential of 324 mV under the same conditions. It was reported that this hybrid structure has better performance and stability than RP-LSCF and P-LSCF from the strong metal-oxygen sharing and high oxygen-ion diffusion rate due to the phase mixture. In addition, complex perovskites such as double and triple perovskites are being studied in various ways (Kim et al., 2018; Wang et al., 2018; Zhu et al., 2019).

The perovskite/carbon hybrid electrocatalysts were also studied to solve the low electrical conductivity of perovskite (Wu et al., 2018; Lin et al., 2021). Shao et al. reported the in situ-introduced carbon nanotubes (CNTs) on the perovskite surface through chemical vapor deposition (CVD) in order to improve the activity and stability of OER. This electrocatalyst showed superior activity than physically mixed perovskite and carbon black, and a perovskite without carbon.

## Conclusion and outlook

Many studies have been conducted on the water electrolysis system, which is an eco-friendly hydrogen production process. Among them, the development of an electrocatalyst for the OER, which requires more overpotential compared to the HER in the system, is a very important research topic. Perovskite electrocatalysts have been studied for their superior activity through various combinations and active site control. Various combinations of perovskite and electrocatalysts using various methods of synthesis, substitution, and exsolution have been reported. These electrocatalysts can enhance the active site through composition engineering, morphology control, hybridization, and etc., highlighting that the development of perovskite electrocatalysts is limitless. Descriptors that can explain and understand the excellent OER performance of perovskite have been discovered by many researchers. In addition, not only the OER mechanism based on the transition metal in perovskite, but also a new reaction mechanism in which lattice oxygen in perovskite participates in the reaction is being studied, and based on this, new combinations of various perovskite OER electrocatalysts are being developed.

However, there are still problems to be solved. Perovskite is mainly synthesized at high temperature, and therefore the specific surface area of the electrocatalyst is significantly low. It can be considered that the active site area of the electrocatalyst is low, and it is necessary to increase the surface area for higher activity. In addition, there is a problem in that the stability of the electrocatalyst is decreased due to the dissolution problem of metal ions inside of the perovskite. Moreover, critically, the perovskite-based electrocatalysts have a disadvantage in that the electrical conductivity is too low, and larger scale synthesis of them should be proved for practical AEMWE applications based on perovskite OER electrocatalysts. Up to now, there are only reports for synthesizing perovskite-based electrocatalysts at the level of lab scale, but it is not clear whether the perovskite can be synthesized well even at a synthesis level of pilot scale or more. Numerous perovskite electrocatalysts have been published, but methods for preparing perovskite electrocatalysts with better stability/activity should be sought. To this end, a method to improve the activity and stability of the electrocatalyst by making a new active site interface different from the existing mechanism is being studied, and the corresponding descriptor should also be developed.
